# Cost-effectiveness of psychotherapies and pharmacotherapies for mental disorders

**DOI:** 10.1192/j.eurpsy.2023.30

**Published:** 2023-07-19

**Authors:** P. McCrone

**Affiliations:** University of Greenwich, London, United Kingdom

## Abstract

**Abstract:**

Mental health problems have a substantial economic impact across the world. Those who have their problems detected may receive therapies (usually medication or psychological) or who packages of care (for example, early intervention or residential support). Some problems are transient while others will remain across the life course. Costs are closely associated with care received and it may be that costs need to rise in order to provide adequate support. Economic costs also occur due to lost opportunities such as work and leisure. This talk will summarise recent estimates of the cost of mental health problems in a number of countries.

Establishing total costs does not tell us how to use limited resources. This requires the use of economic evaluation methods such as cost-effectiveness and cost-utility analyses. There is a growing evidence base from such evaluations for many psychological and pharmacological interventions and recent key findings will be discussed. The need for long-term, life course approaches to economic evaluation of mental health interventions will be proposed.

**Disclosure of Interest:**

None Declared

